# Abnormal Dynamic Functional Networks in Subjective Cognitive Decline and Alzheimer's Disease

**DOI:** 10.3389/fncom.2022.885126

**Published:** 2022-05-02

**Authors:** Jue Wang, Kexin Wang, Tiantian Liu, Li Wang, Dingjie Suo, Yunyan Xie, Shintaro Funahashi, Jinglong Wu, Guangying Pei

**Affiliations:** ^1^School of Life Science, Beijing Institute of Technology, Beijing, China; ^2^Department of Neurology, Xuanwu Hospital, Capital Medical University, Beijing, China; ^3^Kokoro Research Center, Kyoto University, Kyoto, Japan; ^4^Laboratory of Cognitive Brain Science, Department of Cognitive and Behavioral Sciences, Graduate School of Human and Environmental Studies, Kyoto University, Kyoto, Japan; ^5^Research Center for Medical Artificial Intelligence, Shenzhen Institutes of Advanced Technology, Chinese Academy of Science, Shenzhen, China

**Keywords:** resting-state fMRI, Alzheimer's disease, subjective cognitive decline, dynamic functional connectivity, sensorimotor network, clustering analysis

## Abstract

Subjective cognitive decline (SCD) is considered to be the preclinical stage of Alzheimer's disease (AD) and has the potential for the early diagnosis and intervention of AD. It was implicated that CSF-tau, which increases very early in the disease process in AD, has a high sensitivity and specificity to differentiate AD from normal aging, and the highly connected brain regions behaved more tau burden in patients with AD. Thus, a highly connected state measured by dynamic functional connectivity may serve as the early changes of AD. In this study, forty-five normal controls (NC), thirty-six individuals with SCD, and thirty-five patients with AD were enrolled to obtain the resting-state functional magnetic resonance imaging scanning. Sliding windows, Pearson correlation, and clustering analysis were combined to investigate the different levels of information transformation states. Three states, namely, the low state, the middle state, and the high state, were characterized based on the strength of functional connectivity between each pair of brain regions. For the global dynamic functional connectivity analysis, statistically significant differences were found among groups in the three states, and the functional connectivity in the middle state was positively correlated with cognitive scales. Furthermore, the whole brain was parcellated into four networks, namely, default mode network (DMN), cognitive control network (CCN), sensorimotor network (SMN), and occipital-cerebellum network (OCN). For the local network analysis, statistically significant differences in CCN for low state and SMN for middle state and high state were found in normal controls and patients with AD. Meanwhile, the differences were also found in normal controls and individuals with SCD. In addition, the functional connectivity in SMN for high state was positively correlated with cognitive scales. Converging results showed the changes in dynamic functional states in individuals with SCD and patients with AD. In addition, the changes were mainly in the high strength of the functional connectivity state.

## Introduction

Alzheimer's disease (AD) is a common neurodegenerative disease and is characterized by β-amyloid deposition, pathological tau, and neurodegeneration (Jack et al., 2018). The pathogenesis of the above-related proteins may be influenced by the apolipoprotein E epsilon 4-allele gene (Saeed et al., 2018), family history of AD (Yi et al., 2018), sleep quality (Rasmussen et al., 2018), and dietary habits (Elhaik Goldman et al., 2018). Clinically, patients with AD suffer progressive cognitive impairment until daily life is affected and the process is irreversible (Frisoni et al., 2017). Therefore, more and more researchers investigated drug treatment (Schwarz et al., 2018) or mindfulness training (Smart et al., 2016) on neurodegeneration and cognitive decline based on the preclinical stages of AD. Subjective cognitive decline (SCD) refers to the self-reported experience of memory loss and is regarded as the earliest stage of AD (Taylor et al., 2018). Mild cognitive impairment (MCI) is a clinical stage between normal aging and dementia, the pre-stage of AD after SCD, with a 10–15 annual conversion rate to dementia (Arvanitakis et al., 2019). Individuals have subtle cognitive changes that do not interfere with everyday activities in the MCI period. Previous studies indicate that the predementia stage may last 15–20 years (Villemagne et al., 2013; Jansen et al., 2015), and individuals with SCD have more chance of progression to MCI and further AD dementia (Jessen et al., 2014; Snitz et al., 2018). A previous study has revealed that mismatch negativity neurofeedback may represent an effective treatment for intervention in patients with SCD and the elderly with aging memory decline (Pei et al., 2020). However, the pathology of SCD is heterogeneous and can be associated with gender (Roberts et al., 2012), sleep (Lauriola et al., 2017), gene (Moreno-Grau et al., 2018), and psychological impactors (Wake et al., 2018). A previous study has proved that the apolipoprotein E (APOE) ε4 allele is a genetic risk factor for AD, whereas educational attainments have protective effects against cognitive decline in aging and patients with AD (Li et al., 2021). Thus, the progression from SCD to MCI and AD may need multidimensional analyses, such as clinical cognitive scales (Tabatabaei-Jafari et al., 2018), education level (Xu et al., 2021), structural indexes (Moore et al., 2018), functional indexes (Sun et al., 2016), cerebrospinal fluid (Hays et al., 2018), and cortical glucose metabolism measured by positron emission tomography (PET) (Eliassen et al., 2017).

Structural neuroimaging methods have been applied in the study of neurodegenerative disease (Yue et al., 2018; Liu et al., 2021). Studies on SCD revealed that the cortical atrophy in medial temporal structures (Buckley et al., 2017) and the temporal cortical thickness were associated with the subsequent rate of memory loss (Verfaillie et al., 2018a). Also, graph analysis showed that the random organization of the structural gray matter network was related to cognitive functions in individuals with SCD (Verfaillie et al., 2018b). Compared with cortical changes in gray matter, individuals with SCD also showed the abnormality in white matter measured by diffusion tensor imaging (DTI). Previous studies have proved the disrupted structural brain network pattern in individuals with SCD (Yan et al., 2018). Specifically, the structural damage measured by DTI was associated with the cerebrospinal fluid neurofilament light, a protein biomarker in individuals with MCI (Moore et al., 2018). In addition, a resting-state magnetoencephalography (MEG) study revealed the functional network disruption in individuals with SCD and MCI, compared with NC (Lopez-Sanz et al., 2017a,b).

The resting-state functional magnetic resonance imaging (rs-fMRI) measures blood oxygen level-dependent (BOLD) signals. It has the potential to measure the changes in preclinical AD (Ten Kate et al., 2018). The BOLD signals are the intrinsic property of the brain, and they keep the patterns even during task paradigms. The amplitude of low-frequency fluctuation (ALFF) and fractional ALFF (fALFF) may help detect the underlying pathological mechanism in the AD continuum. ALFF/fALFF measurements of spontaneous or intrinsic brain activity may be useful to characterize the early gradient of physiological alterations in AD (Yang et al., 2018, 2020). Functional connectivity (FC) can measure the intrinsic patterns by calculating the spatial correlations between different brain regions (Fox and Raichle, 2007). An increased correlation was observed between central frequency and cognitive performance in posterior cortical regions in individuals with SCD compared with NC (Xie et al., 2019). A study that combined structural MRI and rs-fMRI showed that graph measures extracted by resting-state FC had the better power for predicting the conversion from MCI to AD compared with cortical thickness extracted by structural MRI (Hojjati et al., 2018). Resting-state FC can be used to reflect individual brain patterns of cognitive behaviors (Finn et al., 2015). A previous study revealed the reduction in metastability of the resting-state BOLD signals of patients with AD from the network level (Alderson et al., 2018). A previous study has revealed the abnormal interactions among large-scale networks, such as the dorsal attention network, the central-executive network, and the default mode network (DMN), in individuals with MCI compared with NC (Chand et al., 2018). Numerous studies constructed the resting-state FC and showed the disruption of DMN in patients with AD, the precuneus/posterior cingulate cortex (Grieder et al., 2018; Yokoi et al., 2018). However, studies above only focus on the time-stable property, not on the time-varying property of the brain in resting state. The brain is complex and variable, and more studies have proved that the changes in FC across time help reveal dynamic changes in brain function. A growing number of fMRI studies have found dynamic FC that captures the time-sensitive information about dynamic brain network (Calhoun et al., 2014; Pervaiz et al., 2020; Barber et al., 2021).

Over the years, MRI-based computer-aided diagnosis has been shown to be helpful for the early prediction of cognitive decline. An increasing number of studies have adopted machine learning for the classification of AD, with promising results (Wong et al., 2021). Resting-state dynamic functional networks are powerful for the classification of MCI (Jie et al., 2018). Many studies focused on the spatially abnormal brain regions or networks. Recently, temporally abnormal states attracted more and more attention. Clustering analysis is one of the important methods for the functional parcellation of cerebrocortical areas (Garcia-Garcia et al., 2018; Ogawa et al., 2018). A previous study used clustering analysis to reveal the clusters of healthy subjects' resting-state networks from the spatial aspect. They clustered the resting-state networks into “primary sensory/motor networks” and “cognitive networks” (Bijsterbosch et al., 2017). Another study clustered the edges into five classes (Kang et al., 2017). The clustering analysis can also be applied to dynamic FC states extraction. Dynamic FC states extraction allowed researchers to find the repetitive patterns or analyze the contribution of different networks at different time points (Preti et al., 2017). A previous study revealed dynamic functional states in the mouse brain and found the complex reorganization of functional networks by sliding window analysis (Grandjean et al., 2017). It has been proved that the dynamic brain network was associated with behavioral state shifts and may be served as the biomarker of related disorders (Kucyi et al., 2017; Shine and Poldrack, 2018). Specific patients may show specific abnormal dynamic states at specific moments (Preti et al., 2017). One study on schizophrenia showed the temporally abnormal state typified by strong connectivity (Damaraju et al., 2014). A previous study clustered the resting-state dynamic FC of patients with AD into five states and showed obviously the low connective state and high connective state (de Vos et al., 2018).

A recent study revealed the normative pathways in FC from the perspective of space (Leming et al., 2019). In this study, we tried to find the normative states from the perspective of time. One study used the *k*-means clustering analysis and found two states in patients with Parkinson's disease and healthy controls. One state is “more frequent and sparsely connected” (similar to low correlation state), and the other state is “less frequent and more strongly interconnected” (similar to high correlation state) (Kim et al., 2017). Similarly, another study also identified two states (strong and weak states) based on healthy subjects' resting-state networks (Choe et al., 2017). Therefore, we assumed that the normative states in terms of time at least included the low and high connective states (de Vos et al., 2018). In addition, a previous study showed that highly connected brain regions behaved more tau burden in patients with AD (Cope et al., 2018). Thus, we assumed that the high connective state is more vulnerable than other states during the progression from SCD to AD.

## Materials and Methods

### Participants

Our dataset was composed of 165 participants, including 65 NC, 41 participants with SCD, and 59 patients with AD. The diagnoses of SCD and AD were completed by experienced neurologists. The standard of diagnosis and inclusion criteria for NC, SCD, and AD were similar to a previous study (Yan et al., 2018). All the participants agreed to and signed the informed consent prior to scanning. This study was authorized by the Medical Research Ethics Committee and Institutional Review Board of XuanWu Hospital (ClinicalTrials.gov identifiers: NCT02353884 and NCT02225964).

All participants completed the Chinese version of the Mini-Mental State Examination (MMSE), the Beijing version of the Montreal Cognitive Assessment (MoCA), and the auditory verbal learning test (AVLT) to evaluate their cognitive capacity. In addition, the AVLT included AVLT-immediate recall (AVLT-I), AVLT-delayed recall (AVLT-D), and AVLT-recognition (AVLT-R).

### MRI Acquisition

Structural MRI and rs-fMRI data were collected using a 3T Siemens Magnetom Trio Tim MRI scanner with a standard head coil. Cushions and headphones were used to minimize movements and scanner noise.

The rs-fMRI data of participants were collected during the eyes-closed and relaxed condition. A standard gradient-echo echo planar imaging sequence was used with repetition time (TR) = 2,000 ms, echo time = 40 ms, flip angle = 90°, field of view = 240 × 240 mm^2^, matrix size = 64 × 64, thickness = 4.0 mm, number of slices = 28, voxel size = 3.75 × 3.75 × 4 mm^3^, and layer interval = 1 mm. Each resting-state scan lasted for 478 s (239 volumes).

Structural T1 data acquisition was same to a previous published study (Yan et al., 2018). T1-weighted high-resolution data were collected using a 3D magnetization-prepared rapid gradient echo (MPRAGE) with repetition time = 1,900 ms, echo time = 2 ms, inversion time = 900 ms, flip angle = 9°, field of view = 224 × 256 mm^2^, matrix size = 448 × 512, no gap, thickness = 1.0 mm, and number of slices = 176.

### The rs-fMRI Data Preprocessing

**Figure 1:**
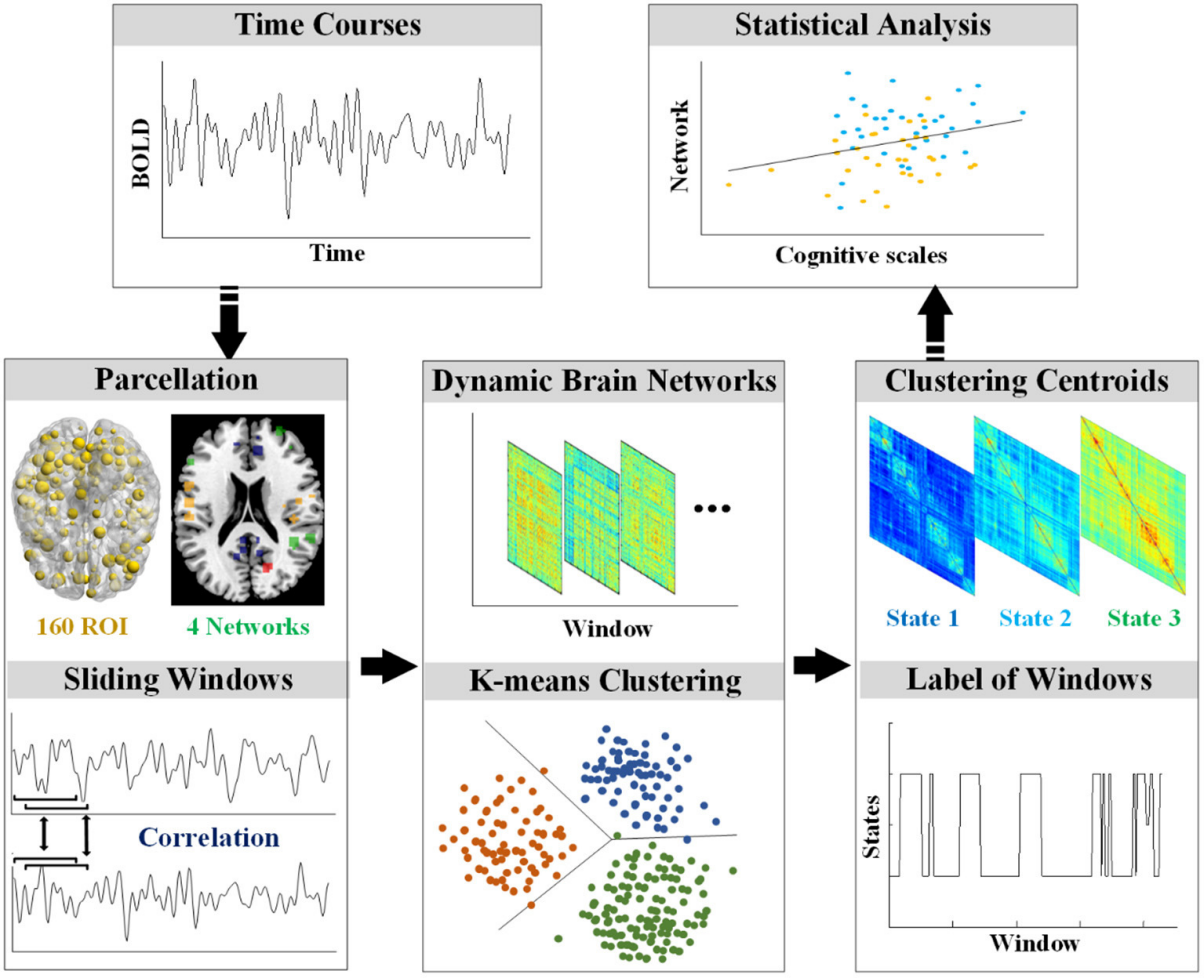
The workflow of processing.

The rs-fMRI data were preprocessed using Statistical Parametric Mapping software (SPM12, https://www.fil.ion.ucl.ac.uk/spm/) and the Graph Theoretical Network Analysis Toolbox for Imaging Connectomics (GRETNA, https://github.com/sandywang/GRETNA). Both SPM and GRETNA (Wang et al., 2015) were implemented in MATLAB (version R2014a, MathWorks, Inc., Natick, MA, USA). The first ten volumes were removed to allow for scanner stabilization and adaption of participants, resulting in 229 volumes. The 229 volumes were slice-timing corrected to allow for timing differences in slice acquisition, realigned to the first volume, co-registered to the corresponding T1 image, normalized to the Montreal Neurological Institute (MNI) template, and resampled to 3 mm^3^. Then, the fMRI data were smoothed with a Gaussian kernel (FWHM = 5 mm) and removed linear trends. In addition, several nuisance signals (six head motion parameters, signals from white matter and cerebrospinal fluid) were regressed out by Friston 24-parameter model (Friston et al., 1996). Furthermore, the time course of each voxel was applied temporal filtering (0.01–0.1 Hz). Participants were excluded if the max absolute translations and rotations of their head motions were larger than 3 mm or 3°. The framewise displacement (FD) was calculated to further control the head motion (Power et al., 2012). After the preprocessing, the data were processed according to the flowchart in Figure 1.

### Computation for Dynamic Brain Networks

To compute the dynamic brain networks, the DOS atlas (Dosenbach et al., 2010) was applied to define the 160 regions of interest (ROIs) of 10 mm diameter spheres. Furthermore, the 160 ROIs of the DOS atlas were grouped into the following four functional networks: the default mode network (DMN), the cognitive control network (CCN), the sensorimotor network (SMN), and the occipital-cerebellum network (OCN) (Tian et al., 2018).

Dynamic FC analysis was examined by the sliding-time window approach. Previous dynamic FC studies showed that the window size should at least be larger than 30 s (Jones et al., 2012). In this study, the sliding window approach segmented the mean time series of each ROI into many 15-TR windows with the size of 30 s and a time interval of 1 TR, resulting in 215 time-windows for each participant. In each window, the weighted FC was defined by calculating Pearson's correlation on the time series of each pair of ROI. To improve the normality of the distribution of Pearson's correlation coefficients, the Pearson's correlation coefficients were transformed into *z*-scores by Fisher's *z* transformation.

### FC State Analysis/Clustering Analysis

Reoccurring FC states in individual dynamic FC were identified using *k*-means clustering algorithm (Kim et al., 2017; Fu et al., 2018). The sklearn (https://scikit-learn.org/stable/) package in Python v3.6 (https://www.python.org/) was used for the realization of the clustering algorithm. In particular, each of the 215 windows was used as a sample; each upper triangular element of the 160 × 160 matrix was used as a feature (12,720 features in total); and the L1 norm (Manhattan distance) was used as a distance function (Fu et al., 2018). The best *k* (number of clusters or states) was determined by the elbow criterion (Vergara et al., 2020), which was computed as the ratio of within-cluster distance to between-cluster distance (Allen et al., 2014). In addition, the *k*-means++ was the initial cluster centers algorithm (Arthur and Vassilvitskii, 2007). To reduce random errors, the algorithm was repeated 10 times for different centroid seeds and chose the best output as the final result. Specifically, the algorithm was performed on NC, individuals with SCD, and patients with AD.

### Group Differences

The differences among states were evaluated by global and local network comparison. For each participant, the mean FC matrix of each state was calculated based on the label of each window after clustering. In global network, the mean value of the mean FC matrix of each state was calculated for the comparison among groups by ANOVA (*p* < 0.05, Bonferroni corrected). In local network, the mean value of FC in each network (CCN, DMN, SMN, and OCN) was calculated for the comparison among groups by ANOVA (*p* < 0.05, Bonferroni corrected). The visualization of FC in the significantly different networks among the three groups was completed by the Circos software (Krzywinski et al., 2009).

### Associations Between Dynamic FC and Cognitive Scales

The associations between dynamic FC and cognitive scales were evaluated by partial Pearson's correlation analyses (*p* < 0.05). As mentioned above, participants underwent the following cognitive scales: MMSE, MoCA, AVLT-I, AVLT-D, and AVLT-R. The mean value of the five cognitive scales was calculated as the independent variable, and the mean value of global FC matrix was the dependent variable for the global association analysis. Age, education, and FD were covariates. Since the differences among groups were the research focus, the local association was evaluated on the specific network in a specific state that showed significant variation by ANOVA.

## Results

### Demographic and Clinical Characteristics

After the control of head motion (the max absolute translations and rotations of head motions were larger than 3 mm or 3°), 150 participants were included in the present research. The demographic and clinical characteristics were detailed in Table 1. No statistical difference (*p* < 0.05) among the groups was found in gender and FD, which suggests that the head motion was controlled well. Statistical difference among groups was found in the age, education, and five cognitive scales.

**Table 1:** Demographic and clinical characteristics.

	**NC**	**SCD**	**AD**	***P*-value**
Gender (M/F)	60 (26/34)	40 (16/24)	50 (17/33)	*p =* 0.605^a^
Age (years)	62.57 ± 8.67	64.90 ± 8.31	70.86 ± 9.87	*p =* 0.000^a^
Education (years)	10.85 ± 5.06	11.65 ± 4.53	8.84 ± 5.65	*p =* 0.027^a^
FD (mm)	0.25 ± 0.12	0.22 ± 0.13	0.27 ± 0.12	*p =* 0.161^a^
AVLT-I	9.23 ± 1.89	8.32 ± 1.92	3.59 ± 1.61	*p < * 0.000^a^
AVLT-D	10.30 ± 2.87	8.95 ± 2.66	1.00 ± 1.62	*p < * 0.000^a^
AVLT-R	12.08 ± 2.59	11.18 ± 2.75	3.66 ± 3.36	*p < * 0.000^a^
MMSE	28.18 ± 2.13	28.05 ± 1.93	16.61 ± 6.17	*p < * 0.000^a^
MoCA	26.15 ± 3.08	25.51 ± 2.73	12.52 ± 5.06	*p < * 0.000^a^

*Note: Values are presented as mean ± SD*.

*NC, normal controls; SCD, subjective cognitive decline; AD, Alzheimer's disease; FD, framewise displacement; AVLT-I, the auditory verbal learning test (AVLT)-immediate recall scores; AVLT-D, AVLT-delayed recall scores; AVLT-R, AVLT-recognition scores; MMSE, the Chinese version of the Mini–Mental State Examination; MoCA, the Beijing version of Montreal Cognitive Assessment*.

a *χ^2^-test*.

b *One-way ANOVA. p < 0.05 was considered significant*.

### Global Differences

**Figure 2:**
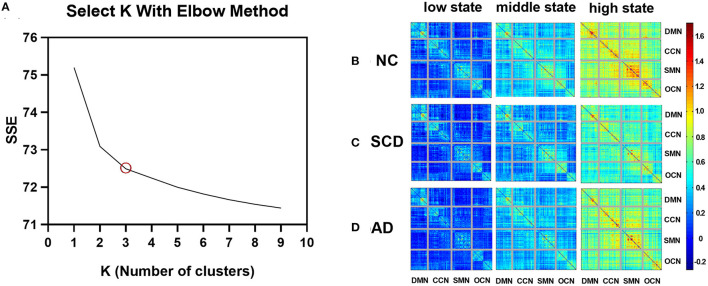
Variations of SSE with *k* and cluster centroids for different states in different groups. **(A)** A line plot between SSE (within-clusters sum of squared errors) vs. *k* (number of clusters). **(B–D)** The three FC states, namely, low state, middle state, and high state, of NC, SCD, and AD groups are shown in **(B–D)**, respectively. The value in the matrix indicates the center of FC measured by Pearson correlation (Fisher's *z*-transformed) in each state.

Taking the elbow criterion and the comparison among groups into the consideration, the best *k* was three. Thus, the dynamic functional brain networks were clustered into three states: the low state, middle state, and high state. The cluster centroids in every group were shown in Figure 2. Specifically, 34 participants were excluded from the following analysis because they showed only one or two states during the whole scan. Thus, the following results were obtained from 116 participants (45 NC, 36 individuals with SCD, and 35 patients with AD).

As described above, each subject has 215 windows across the whole scan, and each window will belong to one of the three states. The number of occurrences of each state in each group is as follows: low state: mean ± standard error (SE) for NC group: 104.133 ± 5.943, for SCD group: 95.778 ± 6.793, and for AD group: 89.657 ± 7.860; middle state: mean ± SE for NC group: 84.444 ± 4.027, for SCD group: 84.111 ± 4.756, and for AD group: 88.486 ± 5.017; and high state: mean ± SE for NC group: 26.422 ± 4.030, for SCD group: 35.111 ± 4.788, and for AD group: 36.857 ± 6.752. In each of the three groups, the low state has the most occurrences, followed by the middle state, and the high state has the least number of occurrences.

**Figure 3:**
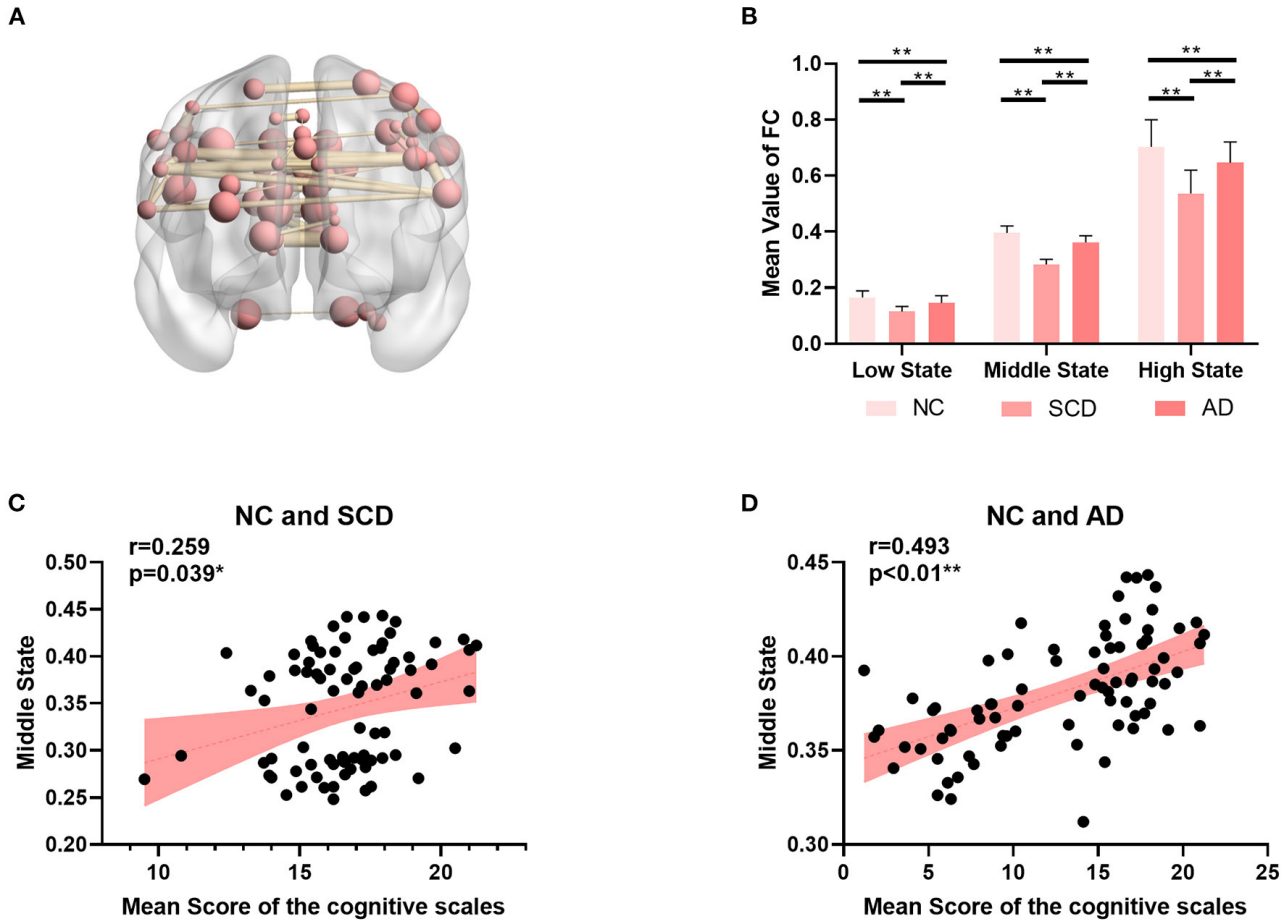
Global differences among NC, SCD, and AD groups. **(A)** The top 5‰ (64/12720) functional connectivity of SMN in middle state. **(B)** The mean value of functional connectivity in each state. The statistically significant *p*-value is 0.01 (**) and Bonferroni corrected. **(C,D)** The plots of the mean value of functional connectivity in middle state vs. mean scores of the cognitive scales were shown in **(C)** for NC and SCD groups and in **(D)** for NC and AD groups. The statistically significant *p*-value is * < 0.05 or ** <0.01.

As shown in Figure 3, the mean value of the FC matrix in each state of each group showed statistical difference (low state: mean ± SE for NC group: 0.17 ± 0.003, for SCD group: 0.11 ± 0.003, and for AD group: 0.15 ± 0.004; *p* < 0.01 for three groups; *p* < 0.01 for NC vs. SCD group; *p* < 0.01 for NC vs. AD group; *p* < 0.01 for SCD vs. AD group; middle state: mean ± SE for NC group: 0.40 ± 0.004, for SCD group: 0.28 ± 0.003, and for AD group: 0.36 ± 0.004; *p* < 0.01 for three groups; *p* < 0.01 for NC vs. SCD group; *p* < 0.01 for NC vs. AD group; *p* < 0.01 for SCD vs. AD group; high state: mean ± SE for NC group: 0.70 ± 0.015, for SCD group: 0.54 ± 0.014, and for AD group: 0.65 ± 0.013; *p* < 0.01 for three groups; *p* < 0.01 for NC vs. SCD group; *p* = 0.014 for NC vs. AD group; *p* < 0.01 for SCD vs. AD group; Bonferroni corrected). The differences suggest that the three states were clustered according to the strength of FC. Furthermore, the mean value of the FC matrix in middle state was positively correlated with the mean scores of the cognitive scales in NC and SCD group [partial correlation, *r*_(76)_ = 0.259, *n* = 81, *p* = 0.039, uncorrected], and NC and AD group [partial correlation, *r*_(75)_ = 0.493, *n* = 80, *p* < 0.01, uncorrected].

### Local Differences

**Figure 4:**
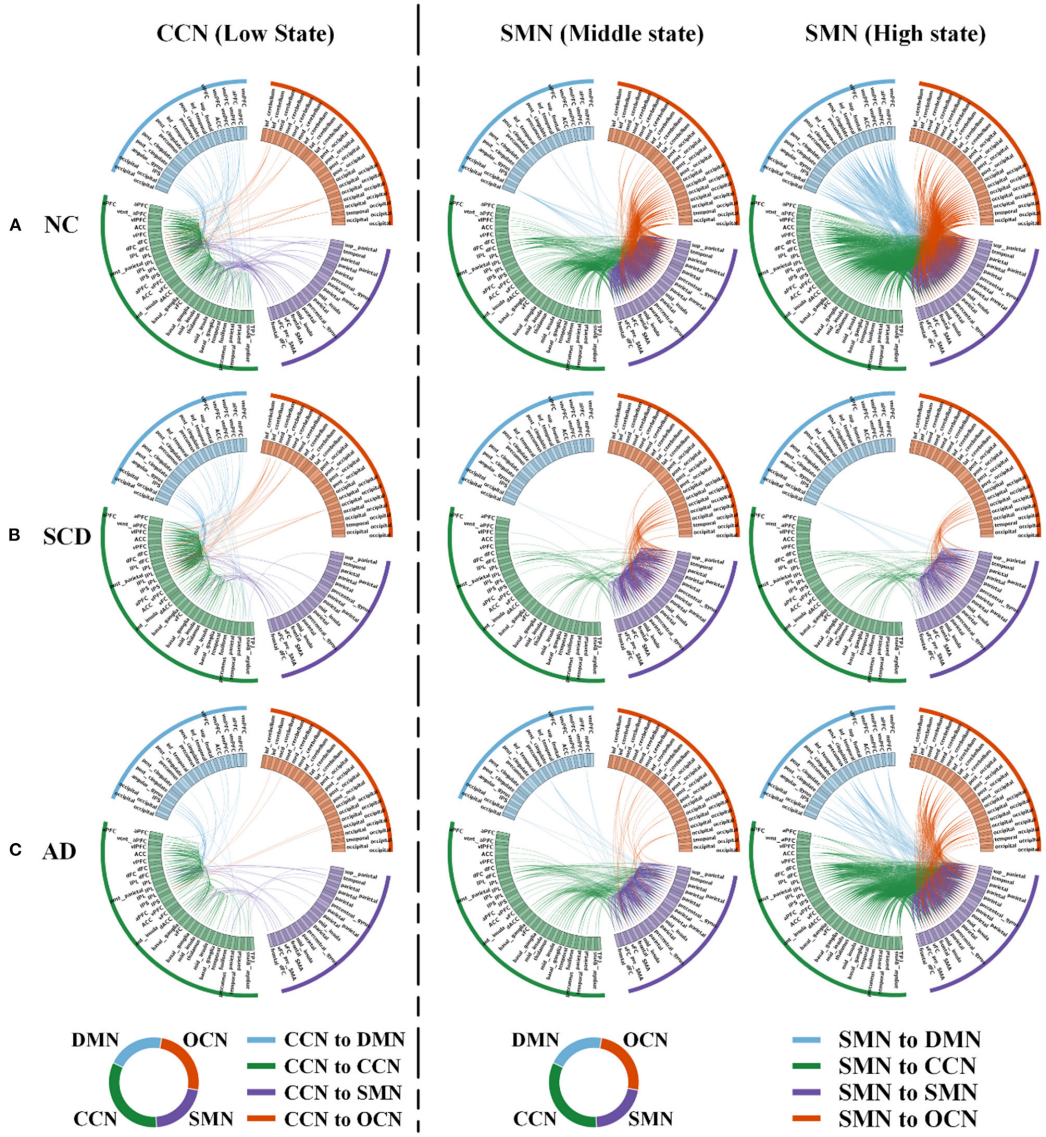
Local differences. The 160 ROIs were arranged along the circle. In each circle, the blue lines indicated the correlations between DMN and CCN (in the first column) or between DMN and SMN (in the second and third columns). The green lines indicated the correlations within CCN (in the first column) or between CCN and SMN (in the second and third columns). The purple lines indicated the correlations between SMN and CCN (in the first column) or within SMN (in the second and third columns). The orange lines indicated the correlations between OCN and CCN (in the first column) or between OCN and SMN (in the second and third columns). **(A–C)** In NC **(A)**, SCD **(B)**, and AD **(C)** groups, low state averaged FC between CCN and the other three networks (i.e., DMN, SMN, and OCN) was visualized. Similarly, middle state and high state averaged FC between SMN and the other three networks (i.e., DMN, CCN, and OCN) were visualized. For the visualization, the correlation coefficients larger than 0.4, 0.5, and 0.8 were shown in the first, second, and third column.

**Figure 5:**
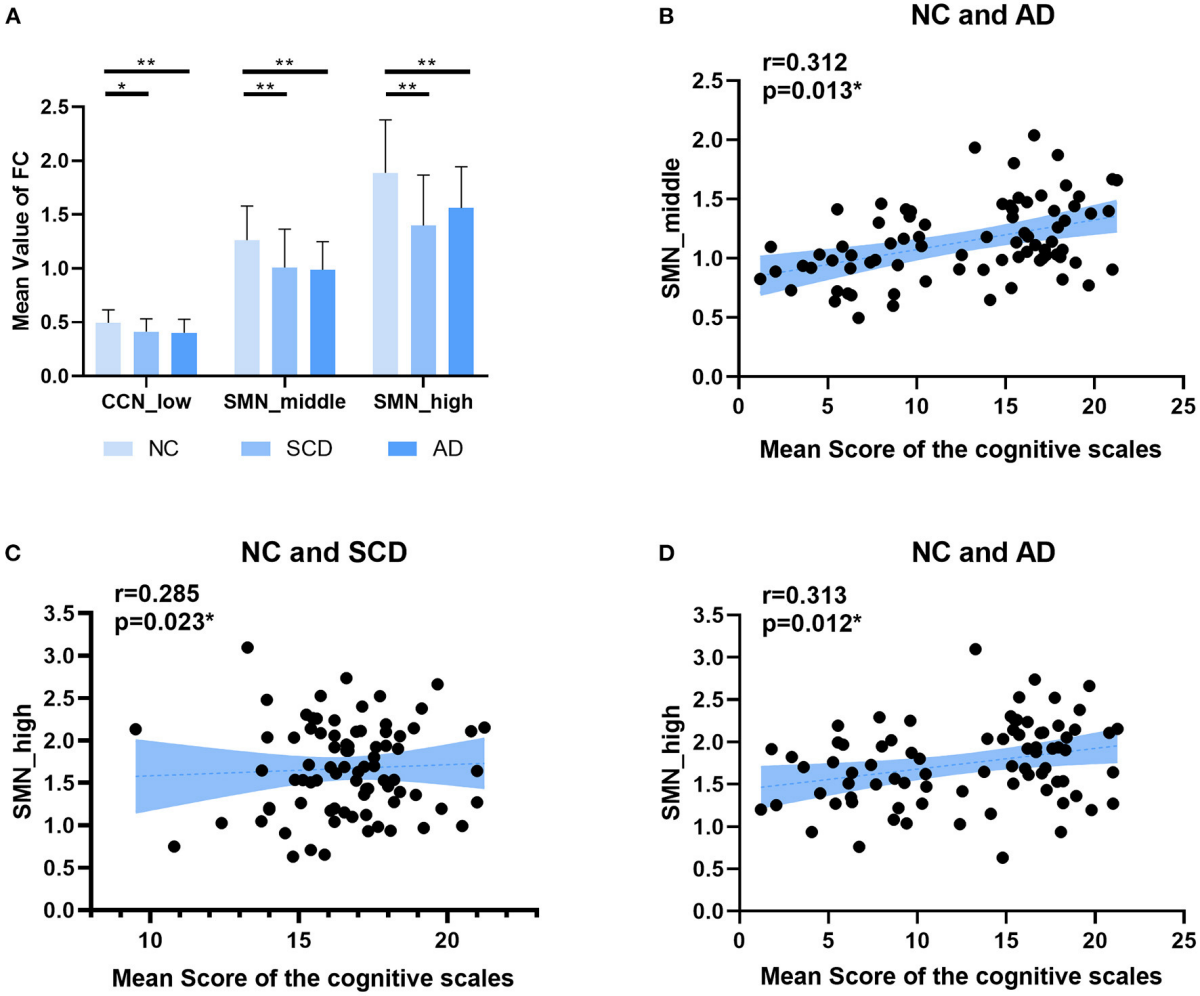
Local differences. **(A)** The statistically significant difference of the mean value of functional connectivity within four networks (i.e., DMN, CCN, SMN, and OCN) among groups (NC, SCD, and AD). The statistically significant *p*-value was 0.05 (*) or 0.01 (**) and Bonferroni corrected. The plots of the mean scores of the cognitive scales vs. the mean value of functional connectivity within SMN in middle state of NC and SCD groups, and SMN in high state of NC and SCD groups and NC and AD groups are shown in **(B–D)** respectively. The statistically significant p-value is (*) 0.05.

For local differences, the mean value of CCN in low state, SMN in middle state, and SMN in high state showed statistical difference both in NC and SCD group and in NC and AD group (CCN in low state: mean ± SE for NC group: 0.49 ± 0.018, for SCD group: 0.41 ± 0.020, and for AD group: 0.40 ± 0.021; *p* = 0.001 for three groups; *p* = 0.01 for NC vs. SCD group; *p* = 0.004 for NC vs. AD group; *p* = 1.00 for SCD vs. AD group; SMN in middle state: mean ± SE for NC group: 1.26 ± 0.047, for SCD group: 1.01 ± 0.059, and for AD group: 0.99 ± 0.044; *p* < 0.01 for three groups; *p* = 0.001 for NC vs. SCD group; *p* = 0.001 for NC vs. AD group; *p* = 1.00 for SCD vs. AD group; SMN in high state: mean ± SE for NC group: 1.89 ± 0.074, for SCD group: 1.40 ± 0.078, and for AD group: 1.56 ± 0.065; *p* < 0.01 for three groups; *p* < 0.01 for NC vs. SCD group; *p* = 0.006 for NC vs. AD group; *p* = 0.393 for SCD vs. AD group; Bonferroni corrected). The visualization of FC between three kinds of network (i.e., CCN in low state, SMN in middle state, and SMN in high state) and other regions was shown in Figure 4. As shown in Figure 5, SCD group and AD group showed fewer connections compared with NC group. Partial Pearson's correlation analyses showed statistical positive correlations (*p* < 0.05) in SMN. Particularly, NC and AD group showed the correlations in both middle state and high state [middle state: partial correlation, *r*_(75)_ = 0.312, *n* = 80, *p* = 0.013; high state: partial correlation, *r*_(75)_ = 0.313, *n* = 80, *p* = 0.012; uncorrected]. However, NC and SCD group showed the correlation only in high state [partial correlation, *r*_(76)_ = 0.285, *n* = 81, *p* = 0.023, uncorrected].

## Discussion

In this study, the reoccurring states of dynamic FC in NC, SCD, and AD were characterized by sliding windows and *k*-means clustering analysis (*k* = 3). The reoccurring states were clustered based on the correlation between each pair of brain regions and included the low state, middle state, and high state in each group. The high state indicated the highly connected within the brain but not always occurred during the rs-fMRI scanning. Based on the three different connected states, changes in global and local dynamic FC were clarified among NC, SCD, and AD groups. For global dynamic FC, each of the three states showed a significant difference in the mean value of the global network among the three groups. The mean value of middle state FC was positively correlated with cognitive scales. The results suggest that the middle state is good for memory and may serve as a preclinical biomarker of AD. For local FC, CCN in low state and SMN in middle state and high state showed significant differences among the three groups. The mean value of SMN in high state was positively correlated with cognitive scales. Taken together, global and local network results suggest that both middle state and high state were the vulnerable dynamic states in the progression of AD.

To the best of our knowledge, this study is the first study to investigate the different connected states based on the resting state dynamic FC in individuals with SCD and patients with AD. In this study, three states were clustered. Some other studies clustered the resting-state FC into five or six states and also revealed similar results. They classified the dynamic brain FC into five states and found that two states belong to the low correlations and three states belong to the high correlations (Plis et al., 2018). A recent study focused on patients with autism spectrum disorder and found four dynamic FC states, including the globally disconnected state (similar to low correlation state), globally hyperconnected state (similar to high correlation state), globally modularized state, and DMN modularized state (Rashid et al., 2018). However, too many clusters would make the contrasts between low state and high state decrease. A previous study clustered the healthy subjects' subject-level spatiotemporal brain networks into 12 clusters by *k*-means clustering analysis (Griffa et al., 2017), and no obvious high state was found. Highly and lowly connected states were common, but the common states would be covered up by too many clusters. Thus, three clusters or states were suitable for characterizing the changes in brain.

The basic goal of this study was to find the changes between NC and individuals with SCD or patients with AD. Compared with NC, a reduction of FC was revealed in individuals with SCD and patients with AD in this study, which was similar to previous studies (Jacobs et al., 2015). Similar changes were found between BOLD and resting EEG coherence (alpha and beta bands). Furthermore, they revealed a positive correlation between blocking γ-aminobutyric acid transmission and resting-state FC (Nasrallah et al., 2017). The reduction of global FC in individuals with SCD and patients with AD indicated the abnormal blocking γ-aminobutyric acid transmission in the progression of AD. A previous study of permutation entropy (PE) showed that AD exhibited lower complexity than did the MCI and NC controls, and the results were related to the results of the regional homogeneity (ReHo) analysis (Wang et al., 2017).

This study not only found the abnormal global dynamic FC but also investigated the changes in different local networks, including CCN, DMN, SMN, and OCN. A recent meta-connectomic analysis revealed that neuropsychiatric disorders, such as AD, were disconnected mainly in the default mode network, frontoparietal network, and sensorimotor network (Sha et al., 2018). The increase of amyloid burden measured by PET in bilateral posterior cingulate cortex showed the reduction of face-name associative memory exam performance in individuals with SCD (Sanabria et al., 2018), and the frontoparietal subnetworks can compensate for mental fatigue-related cognitive decline (Taya et al., 2018). Structural or static FC analysis on patients with MCI and AD has revealed the abnormality in DMN (Vipin et al., 2018). A study based on a multimodal support vector machine (SVM) to investigate the structural and functional connectivity patterns of three stages of AD (i.e., SCD, MCI, and AD) showed that the most discriminating brain regions of AD were mainly located in DMN and subcortical structures (Yan et al., 2019). Dynamic FC analysis showed the state, which was typical of strong correlations between CCN and DMN (Faghiri et al., 2018) and revealed the importance of large-scale networks in the dynamic FC analysis.

Static resting-state FC analysis on early autosomal dominant patients with AD showed the preferential degradation of cognitive networks (such as DMN and dorsal attention networks) over sensorimotor networks (Chhatwal et al., 2018), while, in the present dynamic FC analysis, the abnormality of SMN was more preferential than DMN among the three groups. SMN is one of the commonly identified and replicated resting-state networks and is associated with motor execution and somatosensory components (Hohenfeld et al., 2018). A previous study showed the altered sensorimotor integration in patients with AD reflected by pitch reflex, a behavioral index (Ranasinghe et al., 2017). A DTI study on patients with AD showed abnormal complex graph measures in sensorimotor cortex (Ebadi et al., 2017). A transcranial magnetic stimulation (TMS) and EEG co-registration study revealed the abnormality and the compensatory mechanism of sensorimotor system based on patients with mild AD without motor symptoms (Ferreri et al., 2016). Taken together, the disruptions of SMN may be effective biomarkers in the progression of AD.

In summary, the reoccurring states of resting-state FC, including low state, middle state, and high state, were characterized among NC, SCD, and AD groups by sliding windows and clustering analysis. In local network, SMN in high state showed a statistical difference compared with NC, and it is positively correlated with the mean score of the cognitive scales. Therefore, SMN in highly connected brain states behaved more vulnerable in individuals with SCD and patients with AD. Motor execution and somatosensory function network abnormalities may be related to the early stage of AD. Last but not least, this study has a few limitations that should be considered. We need a follow-up study to evaluate the conversion from SCD to AD and to predict the stability or conversion of individuals with SCD (Bessi et al., 2018). Future studies should shed light on the correlation between high state and pathological proteins in large-scale networks, such as DMN and SMN.

## Data Availability Statement

The raw data supporting the conclusions of this article will be made available by the authors, without undue reservation.

## Ethics Statement

The studies involving human participants were reviewed and approved by the Medical Research Ethics Committee and Institutional Review Board of Xuanwu Hospital. The patients/participants provided their written informed consent to participate in this study.

## Author Contributions

JWa designed the study, analyzed and interpreted the data, and wrote the manuscript. KW and TL analyzed the data and performed the statistical test. LW, DS, and SF edited the manuscript and guided the research. YX performed the acquisition of data. JWu and GP revised the manuscript and supervised the whole research. All authors contributed to the article and approved the submitted version.

## Funding

This work was supported by the National Natural Science Foundation of China (grant numbers U20A20191, 61727807, 82071912, and 12104049), the Beijing Municipal Science and Technology Commission (grant number Z201100007720009), the Shenzhen Peacock Plan (grant number KQTD20180413181834876), the Fundamental Research Funds for the Central Universities (grant number 2021CX11011), and the China Postdoctoral Science Foundation (grant number 2020TQ0040).

## Conflict of Interest

The authors declare that the research was conducted in the absence of any commercial or financial relationships that could be construed as a potential conflict of interest. The reviewer BW declared a past co-authorship with the author LW.

## Publisher's Note

All claims expressed in this article are solely those of the authors and do not necessarily represent those of their affiliated organizations, or those of the publisher, the editors and the reviewers. Any product that may be evaluated in this article, or claim that may be made by its manufacturer, is not guaranteed or endorsed by the publisher.
